# Poly(ethylene glycol) based nanotubes for tuneable drug delivery to glioblastoma multiforme†

**DOI:** 10.1039/d0na00471e

**Published:** 2020-08-24

**Authors:** Majed Alghamdi, Filippo Chierchini, Dimitri Eigel, Christian Taplan, Thomas Miles, Dagmar Pette, Petra B. Welzel, Carsten Werner, Wenxin Wang, Catia Neto, Mark Gumbleton, Ben Newland

**Affiliations:** School of Pharmacy and Pharmaceutical Sciences, Cardiff University King Edward VII Avenue Cardiff CF10 3NB UK newlandb@cardiff.ac.uk; Leibniz-Institut für Polymerforschung Dresden, Max Bergmann Center of Biomaterials Dresden Hohe Straße 6 D-01069 Dresden Germany; Charles Institute of Dermatology, School of Medicine, University College Dublin Ireland; School of Pharmacy, King Abdulaziz University Jeddah 21589 Saudi Arabia

## Abstract

Glioblastoma multiforme (GBM) is the most aggressive type of malignant brain tumour, which is associated with a poor two-year survival rate and a high rate of fatal recurrence near the original tumour. Focal/local drug delivery devices hold promise for improving therapeutic outcomes for GBM by increasing drug concentrations locally at the tumour site, or by facilitating the use of potent anti-cancer drugs that are poorly permeable across the blood brain barrier (BBB). For inoperable tumours, stereotactic delivery to the tumour necessitates the development of nanoscale/microscale injectable drug delivery devices. Herein we assess the ability of a novel class of polymer nanotube (based on poly(ethylene glycol) (PEG)) to load doxorubicin (a mainstay breast cancer therapeutic with poor BBB permeability) and release it slowly. The drug loading properties of the PEG nanotubes could be tuned by varying the degree of carboxylic acid functionalisation and hence the capacity of the nanotubes to electrostatically bind and load doxorubicin. 70% of the drug was released over the first seven days followed by sustained drug release for the remaining two weeks tested. Unloaded PEG nanotubes showed no toxicity to any of the cell types analysed, whereas doxorubicin loaded nanotubes decreased GBM cell viability (C6, U-87 and U-251) in a dose dependent manner in 2D *in vitro* culture. Finally, doxorubicin loaded PEG nanotubes significantly reduced the viability of *in vitro* 3D GBM models whilst unloaded nanotubes showed no cytotoxicity. Taken together, these findings show that polymer nanotubes could be used to deliver alternative anti-cancer drugs for local therapeutic strategies against brain cancers.

## Abstract 2

Soft and flexible poly(ethylene glycol)-based nanotubes load doxorubicin for controlled release to glioblastoma multiforme.

## Graphical abstract

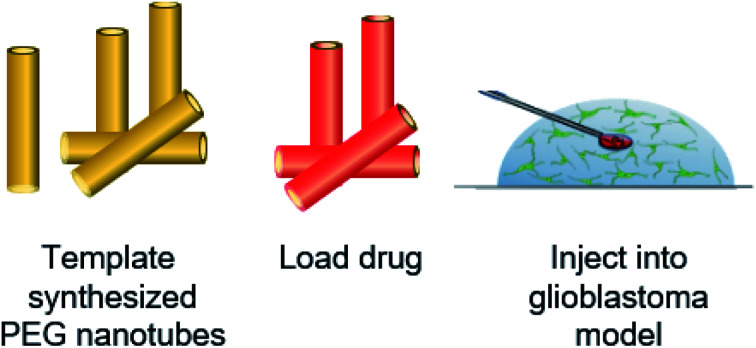

## Introduction

Glioblastoma multiforme (GBM) is classified as a grade IV glioma by the World Health Organisation (WHO)^1^ and is the most common and aggressive malignant brain tumour. Current therapeutic strategies result in a median survival rate that is less than two years.^2^ Symptoms of GBM include thromboembolism, seizures, headaches, vision problems, memory loss and nausea/vomiting.^3^ The current treatment of newly diagnosed GBM patients utilises surgical resection of the tumour to relieve the symptoms, decrease the bulk of the tumour and to aid the diagnosis. However, surgery is only applicable to 65–75% of patients (depending on the position of the tumour).^4^ Following surgery, patients typically receive radiotherapy for 6 weeks, together with an adjuvant chemotherapeutic treatment of an alkylating agent, temozolomide (TMZ).^2^ However, GBM is associated with a high rate of recurrence due to the infiltrative nature of GBM.^5^ Despite the recent advances in surgery, radiation and chemotherapy, the prognosis has not significantly improved and thus development of new therapies is needed.

The rationale for local delivery of chemotherapeutic agents to GBM include decreasing the adverse effects of systemically administered therapeutics, targeting the tumour site, and expanding the range of chemotherapeutic options.^6^ Local delivery can be achieved by direct insertion of a drug or drug delivery system into the GBM surgical resection cavity or *via* injection into inoperable GBM.^7,8^ Local delivery of chemotherapeutic agents also holds the potential to increase the concentration of the drug at the tumour site without a corresponding increase in the systemic concentration and harmful off-target effects.^9^ GBM is locally infiltrative and recurrence is reported to often occur within 2 cm of the original tumour location (80–90% of the cases).^5,10^ Local delivery of chemotherapeutic agents to the site of the resected tumour may therefore have a better chance of killing peripheral non-resected tumour cells. In addition, local delivery bypasses the blood brain barrier (BBB) thus enabling the utilisation of therapeutics that cannot cross the BBB with sufficient efficiency.^11^ For example, direct intracranial injection *via* convection enhanced delivery has allowed the administration of carboplatin,^12^ a hydrophilic drug that does not freely cross the BBB.^13^ Local drug delivery strategies therefore vastly expand the range of possible chemotherapeutic agents available for GBM therapies. For example, a drug such as doxorubicin (a mainstay therapeutic for many breast cancers) offers exciting prospects, since it has shown much better cytotoxic potency than TMZ against GBM cells *in vitro*.^14^

Injectable drug delivery systems, which control the release of a drug at the tumour site, offer the possibility for a single intervention in conjunction with radiotherapy and other chemotherapeutics. Whilst Gliadel® wafers have been commonly used to release carmustine into the GBM resection cavity,^15^ these wafers are 1.45 cm in diameter making them unsuitable for applications requiring injection (*e.g.* GBM without resection). Injectable drug delivery systems may therefore hold advantages over wafers, not just in terms of range of utility, but also in terms of better drug penetration depth and avoiding implant dislodgements.^9,16–18^

We hypothesised that by combining the high GBM cytotoxicity of doxorubicin with an injectable polymer nanotube drug delivery system, we could achieve sustained drug release with high therapeutic potential. It should be noted that the use of doxorubicin for this nanotube-based drug delivery system also holds other potential advantages. First, the drug has a different mechanism of action from TMZ,^19^ thus potentially avoiding the chemoresistance pathway of TMZ (which is very common in GBM^20^). Second, the drug is reported to have radio-sensitisation properties,^21–23^ which may aid concurrent radiotherapy. Finally, the drug has a favourable chemical dissociation charge for electrostatic binding to the drug delivery system.^24^

Nanotubes are nanoscale hollow tubes with a high aspect ratio. Carbon nanotubes have been heavily investigated for applications in the delivery of therapeutics, including delivery to the central nervous system.^25–27^ However, certain types of nanotubes may exhibit asbestos-like toxicity brought about by their rigidity and high aspect ratio.^28^ Polymer nanotubes, synthesized in a sacrificial template, offer an exciting alternative to carbon nanotubes for drug delivery applications.^29^ A wide range of chemical compositions can be investigated, each conferring different physical properties and differing cytotoxicity profiles.^24,30,31^ Herein, our aim was to investigate the use of poly(ethylene glycol) (PEG) based nanotubes for doxorubicin delivery to GBM cells in culture. Furthermore, we aimed to tailor the drug loading and the release profile *via* simple modifications to the nanotube synthesis procedure.

## Experimental

### Materials and methods

#### Synthesis and characterisation of polymer nanotubes

**Fig. 1:**
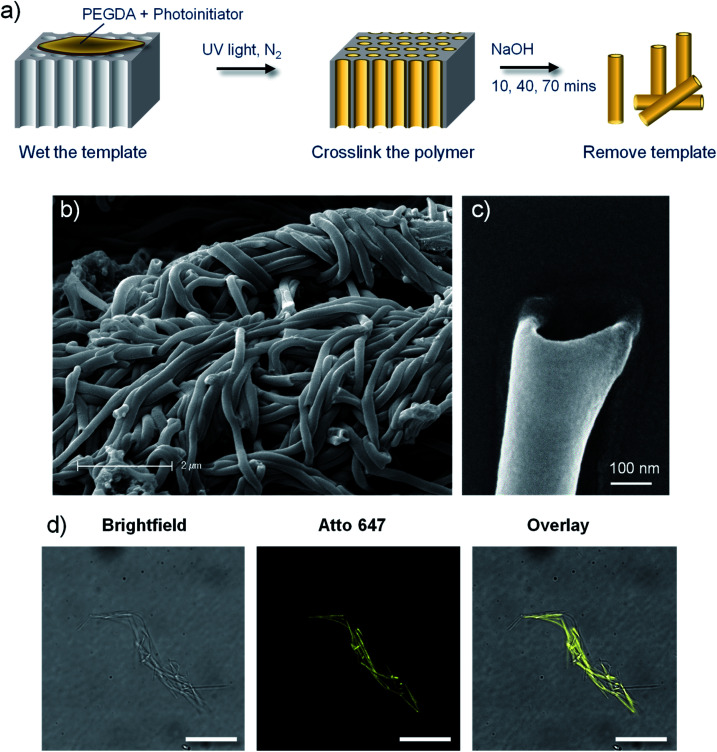
Poly(ethylene glycol) (PEG) based polymer nanotubes *via* template mediated synthesis. (a) Schematic depiction of the polymer nanotube synthesis procedure. Scanning electron microscope images of bundles of PEG nanotubes (b) and the end of an individual nanotube (c). (d) Fluorescent microscope imaging of Atto 647 labelled nanotubes with corresponding brightfield image and subsequent overlay (scale bar represents 20 μm).

All reagents and solvents were purchased from Sigma unless otherwise stated. A schematic depiction of the nanotube synthesis process is provided in Fig. 1a. The polymer nanotubes were synthesised *via* a modification of the previously developed protocol.^24^ Briefly, a monomer solution was prepared by dissolving poly(ethylene glycol)diacrylate (*M*_w_ = 575 g mol^−1^) in acetone (5% wt per vol) together with the photoinitiator 2-hydroxy-2-methylpropiophenone (HMPP) (5 : 1 molar ratio of diacrylate to photoinitiator). To fluorescently label the nanotubes, 10 μg of maleimide functionalized Atto dye (either Atto 463, Atto 565 or Atto 467 (ATTO-TEC GmbH)) was added to 1 mL of the monomer solution. 100 μL of the monomer solution were spread evenly across both sides of an Anodisc™ anodized aluminium oxide (AAO) membrane (Whatman® 200 nm pore size and 47 mm disc diameter, pore depth = 60 μm). The monomer infiltrated AAO membrane was then flushed with nitrogen for two minutes and crosslinked by exposing it to UV light for two minutes (Köhler Technik lamp, UV wavelength = 365 nm, intensity = 1800 μW cm^−2^).

To yield the free PEG based nanotubes, the AAO membrane was dissolved in sodium hydroxide solution (6 mL, 1 M) for 10, 40 or 70 minutes using an ultrasonic bath XUBA1 (Grant) including 30 seconds of probe sonication (Qsonica Sonicator Q55, Fischer Scientific). The standard dissolution protocol used was 40 minutes long, but shorter and longer times were tested to evaluate the effect of dissolution time on drug loading. For all cell studies, PEG nanotubes produced with 40 minutes dissolution time were used. The yellow-white dispersion obtained was washed twice with 1 M sodium hydroxide (1 mL) by centrifugation (13 400 rpm, Minispin (Eppendorf)) and replacement of the supernatant. This was followed by three washes with water and two washes in methanol before subsequent drying under laminar flow.

Scanning electron microscopy (SEM) was carried out using a Zeiss LEO 1525 equipped with GEMINI field emission column with an accelerating voltage of 10 kV. Samples were re-suspended in methanol and a drop was dried on a round glass cover slip attached to an aluminium stub *via* carbon bi-adhesive tape. Prior to analysis, the samples were sputtered with chromium at 100 mA (24 seconds) (Quorum Q150T).

Fluorescence microscopy was carried out using a SP5 laser scanning confocal microscope (Leica). Fluorescently labelled and/or doxorubicin loaded nanotubes were re-suspended in phosphate buffered saline (PBS) at a concentration of 10 μg mL^−1^ and images were acquired using 100× objective lens and 4× digital zoom.

Raman spectroscopy was performed using an Alpha P spectrometer (Bruker) equipped with a DGTS detector and ATR accessory. Pellets of dried nanotubes were investigated in comparison to a crosslinked PEGDA hydrogel (synthesised as described previously^32^) to act as a reference without sodium hydroxide exposure.

### Cell culture

Primary human astrocytes, isolated from the cerebral cortex (ScienCell Research Laboratories) were cultured in astrocyte medium (ScienCell Cat#1801). The human U-87 glioblastoma multiforme cell line and the human U-251 glioblastoma astrocytoma cell line were kind gifts from Professor Johan Bengzon (Lund University) and were cultured in Dulbecco's Modified Eagle Medium (DMEM) (ThermoFisher Cat#31966021) supplemented with 10% fetal bovine serum (FBS, Hyclone ThermoFisher) and 1% penicillin/streptomycin (PS, Life Technologies). Rat C6 glial tumour cells were purchased from Sigma Aldrich (Cat#92090409) and cultured in DMEM/F-12 Ham media (ThermoFisher Cat#11330032) supplemented with 10% FBS and 1% PS. Cells were cultured at 37 °C with 5% CO_2_ using standard cell culturing techniques.

### Cell viability analysis of the nanotubes

Astrocytes were seeded at a density of 10 000 cells per well in a 96-well plate and left overnight to attach. Polymer nanotubes and MWCNTs (Sigma Aldrich, *D* × *L* 110–170 nm × 5–9 μm Cat#659258), were washed in ethanol and dried under laminar flow, before resuspension in cell culture medium for a serial dilution from 120 μg mL^−1^ to 7.5 μg mL^−1^. Medium was removed from the cells and replaced with the appropriate nanotube suspension. Control wells received medium without nanotubes. After incubation for 24 hour or 72 hours, the plates were analysed using a PrestoBlue assay (ThermoFisher, 10% diluted in cell culture medium) according to the manufacturer's protocol. PrestoBlue solution was added to empty wells to act as a blank. Fluorescence was measured using a Tecan GENios plate reader (Tecan), and the cell viability of the test conditions was calculated by normalising to the metabolic activity of the untreated control cells (termed 100% viable). Experiments were carried out in quadruplicate with an average being taken (*n* = 4) and error bars on the graphs plotted represent the standard deviation. Representative light microscope images were taken using an (Olympus IX73) microscope at the end of the test period.

### Analysis of drug loading/release characteristics of the nanotubes

The drug loading capacity of the three different polymer nanotubes (10, 40, or 70 minutes dissolution time – termed PEG-10, PEG-40 or PEG-70, respectively) was evaluated in comparison to MWCNTs. 120 μg of each type of nanotube, were suspended in 1 mL of doxorubicin (LC Laboratories) dissolved in water (containing 0.1% dimethyl sulfoxide (DMSO, Sigma)) at the following concentrations 1.5, 3, 6, 12, 24, 48, and 96 μg mL^−1^, and incubated at room temperature in the absence of light for 72 hours. Doxorubicin solutions without nanotubes were incubated as above to control for doxorubicin degradation.^33^ After incubation, the suspensions were centrifuged for 3 minutes at 13 400 rpm and the concentration in the supernatant was determined *via* UV absorbance at 490 nm (LT-5000 MS plate reader, LabTech) in comparison to the doxorubicin references using a standard curve. The percentage of doxorubicin loaded to the nanotubes was calculated by subtracting the doxorubicin remaining in the supernatant after loading from the doxorubicin in the reference samples (total doxorubicin). This value was then divided by the total doxorubicin and multiplied by 100. This method therefore measures the doxorubicin depletion in the solution due to uptake by the nanotubes.

To analyse the drug release profile from the nanotubes, 120 μg of the different nanotube types were loaded with 96 μg of doxorubicin (in 1 mL water (0.1% DMSO)) at room temperature, in the absence of light, for 72 hours. This represents a drug to nanotube weight ratio of 0.8 : 1 and is passed the saturation point of all nanotube types tested (where no more doxorubicin can be loaded to the nanotubes). At time zero the nanotubes were centrifuged at 13 400 rpm and the supernatant was removed. Drug release was measured from these nanotubes by re-suspending them in 1 mL of PBS and incubating them at 37 °C in the absence of light. Complete removal and replacement of PBS was repeated at subsequent time points, retaining the collected supernatants at −20 °C for concentration analysis (*via* UV absorbance as described above) at the end of the 21 day period. This experiment was performed five times (*n* = 5). Average values were plotted with error bars representing the standard deviation.

### Loading the nanotubes for cytotoxicity experiments

For all subsequent studies PEG nanotubes produced with a 40 minute dissolution time (PEG-40) were used. For all cell culture experiments, a drug to nanotube weight ratio of 0.08 : 1 was used which was below the saturation point (*i.e.* all doxorubicin is loaded to the nanotubes). The reason this ratio was chosen, instead of the 0.8 : 1 used for saturating the nanotubes, is because it means that no free doxorubicin is contained within the medium prior to the cell experiments. Therefore, cytotoxicity shown must be due to the released drug. In addition, this loading ratio also allows comparisons to be drawn with previous experiments.^24^ Doxorubicin loading was carried out as described above using 120 μg of nanotubes (PEG-40 or MWCNT) loaded with 9.6 μg of doxorubicin. After 72 hours at room temperature, the nanotubes were washed twice in the appropriate cell culture medium for the experiment (by centrifugation and removal/replacement of the supernatant) and resuspended at the appropriate concentration for a subsequent dilution series.

### Efficacy testing of drug delivery: 2D culture

C6, U-251 and U-87 cell lines were seeded at a density of 10 000 cell per well in a 96-well plate and left overnight to adhere. The medium was replaced with 100 μL of medium containing nanotubes at concentrations ranging from 7.5 to 120 μg mL^−1^ of either unloaded or doxorubicin loaded nanotubes. Cell viability was analysed, as described above, after 1 and 3 days of incubation with the nanotubes. Experiments were performed in quadruplicate.

### Injection feasibility and cytotoxicity analysis of unloaded *vs.* drug loaded polymer nanotubes in 3D culture

C6 glioma cells were seeded in Matrigel™ (BD Biosciences) at a concentration of 500 cells per μL of Matrigel. Firstly, 15 μL of Matrigel devoid of cells was pipetted as a droplet onto the bottom of wells in a 24-well plate and allowed to polymerise at 37 °C for 15 minutes before another 15 μL of Matrigel containing cells (1000 cells per μL) was placed on top of the polymerised Matrigel. This two-step process prevents cell migration out of the droplet on the surface of the tissue culture plastic. This was left for a further 30 minutes before the addition of 1 mL of medium per well and incubation at 37 °C overnight. PEG nanotubes (either unloaded or doxorubicin loaded as described above) were resuspended in cell culture medium to a concentration of 1675 μg mL^−1^ and 4 μL was administered into the centre of the cell/Matrigel droplet using a 30-gauge stainless steel cannula connected by fine polyethylene tubing to a microsyringe (50 μL, Hamilton) (final concentration of 7.5 μg mL^−1^). PrestoBlue analysis was carried out as above but using a longer incubation time of 3 hours to allow diffusion of PrestoBlue through the Matrigel to the cells. Light microscope imaging of the droplet was performed post injection (to confirm correct positioning) and at the final time point. Confocal microscope live/dead imaging was performed 3 days post-injection using calcein AM solution (PromoKine) and propidium iodide (Sigma) as previously described.^34,35^ Experiments were performed in quadruplicate.

### Analysis of polymer nanotube mediated drug delivery to glioblastoma multiforme spheroids

Glioblastoma Multiforme spheroids were prepared by seeding U-87 cells in round-bottom ultra-low adherence 96-well plates (COSTAR) at a concentration of 1000 cells per well, followed by centrifugation at 300*g* for 1 minute. To allow spheroid formation, the cells were incubated at 37 °C with 5% CO_2_ for 3 days. Polymer nanotube dispersions were prepared as described above for the 2D cell culture assays (unloaded and doxorubicin loaded), but at double the final concentration. They were added to the spheroids, by removing half the cell medium and replacing with medium containing the nanotubes. Light microscopy (Leica DMi1 inverted microscope) was used, together with ImageJ software, to determine the spheroid diameters after 0, 1, 3, 7 and 14 days of incubation. PrestoBlue analysis was carried out after 7 and 14 days using the method outlined above. Experiments were performed with six replicates. The feasibility of direct injection into spheroids was performed using the doxorubicin-loaded nanotubes as described above, at a final concentration (once injected, including the medium surrounding the spheroid) of 15 μg mL^−1^. Glass capillaries were pulled to an approximate outside diameter of 250 μm and connected to the Hamilton syringe/polyethylene tubing as used above. The medium was removed from the wells containing the spheroids and a syringe driver (HLL Landgraf Laborsysteme, Germany) was used to inject 0.25 μL of nanotube suspension or free doxorubicin into the spheroid at a rate of 1 μL per minute. This was performed under a stereo microscope (SE4, Leica) using an eyepiece camera for imaging. Fluorescence microscopy was performed to visualise the injected nanotubes (EVOS M7000 Imaging System) and PrestoBlue analysis was performed as above four days post-injection. Experiments were performed with four replicates.

### Statistical analyses

Data was analysed using GraphPad Prism 6.07 (GraphPad) software. The cell viability of astrocytes was analysed *via* a two-way ANOVA (treatment and dose) for each time point separately with a Sidak post hoc comparisons test. The 2D cell viability studies were analysed using a two-way ANOVA (treatment and dose) for each time point separately with Tukey's post hoc multiple comparisons test. Finally, the 3D cell viability studies were analysed using a one-way ANOVA with Tukey's post-hoc multiple comparisons test. Error bars represent the standard deviation throughout, and an asterisk denotes statistical significance with *P* ≤ 0.05. (Note – asterisks omitted from the graphs of two-way ANOVA analyses for all concentrations below 120 μg mL^−1^ for clarity).

## Results and discussion

### Polymer nanotube synthesis and characterisation

Polymer nanotubes comprised of poly(ethylene glycol) were successfully synthesised *via* a template synthesis procedure. Template synthesized nanomaterials have been produced from starting materials such as metals,^36,37^ nucleic acids,^38^ polysaccharides,^39^ proteins,^40^ and synthetic polymers like polypyrrol^41^ and polystyrene^42^ Such a synthesis strategy gives tight control over the diameter of the resulting nanotubes.^43^ A schematic depiction of the synthesis process is shown in Fig. 1, whereby photopolymerization was used to crosslink PEGDA within the ∼200 nm diameter pores of an anodised aluminium oxide (AAO) membrane. The resulting PEG based nanotubes were released from the template *via* dissolution of the AAO membrane in sodium hydroxide.

Scanning electron microscope (SEM) images show how flexible the nanotubes are, allowing them to deposit over and around each other, with open pore ends on at least one end of the nanotube (Fig. 1b and c). SEM analysis of the PEG nanotubes highlights the uniformity of their diameter which contrasts the large variability exhibited by the multiwalled carbon nanotubes (MWCNT) used as control nanotubes for subsequent studies (ESI Fig. S1†). By adding a maleimide functionalised fluorescent dye to the monomer solution, fluorescently labelled nanotubes could be synthesized (Fig. 1d) provided that the dye's absorption wavelength did not overlap with the wavelength of UV light used for photopolymerization (365 nm). This allowed detection of differently labelled nanotubes (Atto 463, 565 or 647) by fluorescence microscopy (Fig. S2†).

### Doxorubicin loading and release

**Fig. 2:**
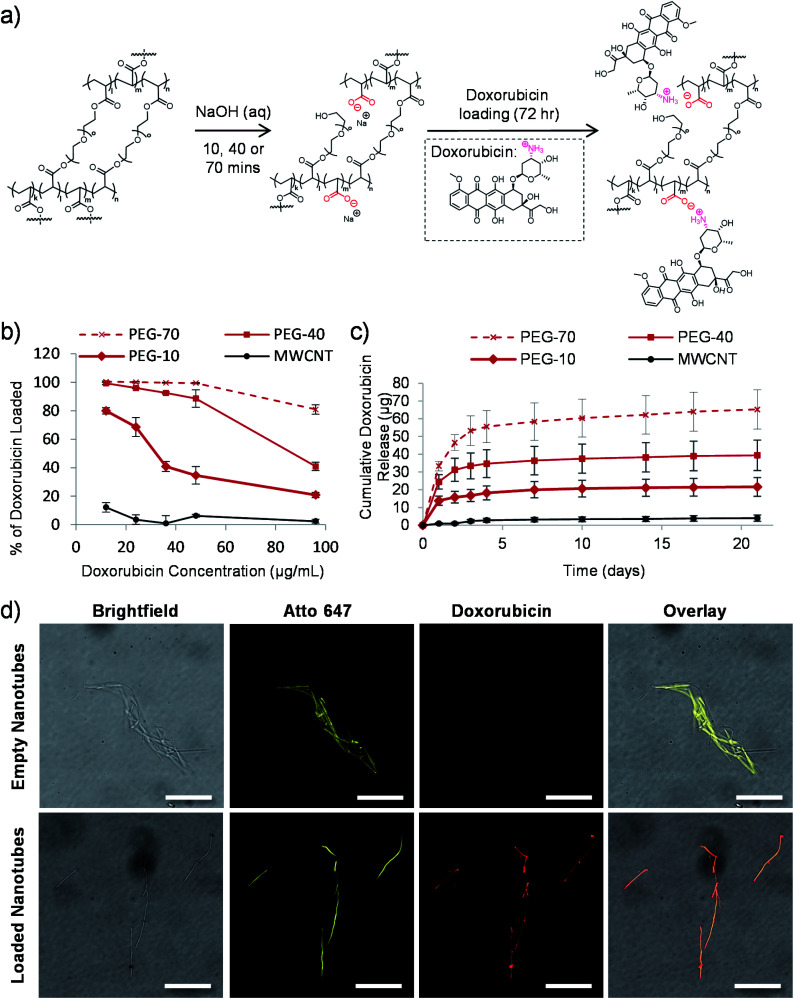
Tuneable drug loading to the nanotubes. (a) Schematic depiction of the proposed mechanism of carboxylic acid group formation in the nanotube structure *via* the sodium hydroxide mediated template dissolution step for either 10, 40 or 70 minutes. These negatively charged groups subsequently drive the electrostatic loading of doxorubicin *via* its amino group. (b) PEG nanotubes removed from the template for either 10, 40 or 70 minutes (termed PEG-10, PEG-40 or PEG-70 respectively) were loaded with varying concentrations of doxorubicin to determine the maximum drug/nanotube loading ratio (saturation point) (*n* = 4). MWCNT were used for a comparison. (c) Time course of doxorubicin release from saturated nanotubes (*n* = 4). (d) Fluorescent microscope images of nanotubes showing that empty nanotubes show no fluorescence at 480 nm excitation, whereas for doxorubicin loaded nanotubes the intrinsic fluorescence of doxorubicin overlaps with that of the Atto 647-labelled nanotube (scale bars represent 25 μm).

We previously hypothesized that dissolution of the AAO membrane, to release the nanotubes from the template, would result in partial cleavage of the ethylene glycol chains present in PEG based nanotubes, thus introducing carboxylic acid groups on the nanotube surface.^24^ As doxorubicin contains a primary amine group which can be protonated (p*K*_a_ value of 8.2),^44^ reversible electrostatic interaction with the nanotubes was envisaged. Fig. 2a shows a schematic depiction of the potential mechanism of sodium hydroxide induced cleavage of PEG chains together with the electrostatic interaction with doxorubicin. We hypothesised that by varying the template dissolution time (and hence exposure time to sodium hydroxide) we could vary the degree of carboxylic acid functionalisation, which could alter the doxorubicin uptake/release properties of the nanotubes. Raman spectroscopy analysis of nanotubes exposed to sodium hydroxide for 10, 40 and 70 minutes (termed PEG-10, PEG-40 and PEG-70 respectively) showed corresponding increases in peak height at 1580 cm^−1^, indicative of increasing carboxylic acid functionalisation (Fig. S3†). Furthermore, incubation of the three types of nanotubes with increasing concentrations of doxorubicin did indeed highlight differences in the loading capabilities of these nanotubes (Fig. 2b). As predicted, PEG-70 showed the highest doxorubicin uptake, removing all the doxorubicin from solutions up to a concentration of 48 μg mL^−1^ (0.4 : 1 doxorubicin to nanotube weight ratio). PEG-40, the standard nanotube used for all subsequent studies, also showed highly efficient doxorubicin uptake, removing all drug from a 12 μg mL^−1^ concentration (0.1 : 1 doxorubicin to nanotube weight ratio). PEG-10 nanotubes were unable to remove all the doxorubicin from the solutions tested but still showed higher drug uptake than the multi-walled carbon nanotube (MWCNT) controls.

To analyse the drug release profile from the nanotubes, all four types were loaded with doxorubicin at a drug/nanotube ratio of 0.8 : 1, *i.e.* above the saturation ratio determined above. After washing off the excess drug, release was analysed over a period of 21 days as shown in Fig. 2c and S4.† Most of the drug was released over the first seven days with a small amount released over the following two weeks (3 μg (7% of loaded drug)). This was true for the three types of PEG nanotubes, with PEG-70 releasing more (as they were loaded with more doxorubicin from the loading solution).

Using fluorescently labelled (Atto-647) PEG nanotubes, the doxorubicin uptake could also be visualized by laser scanning confocal microscopy. Fig. 2d shows overlaid images of the intrinsic fluorescence of doxorubicin with the fluorescently labelled nanotubes, showing homogeneous doxorubicin uptake along the nanotube length.

### PEG nanotubes caused no reduction in cell viability

**Fig. 3:**
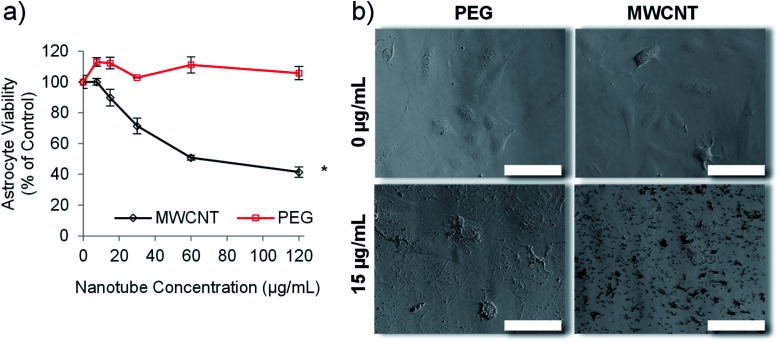
Empty PEG nanotubes show significantly lower toxicity than MWCNT. The viability of human astrocytes, as determined *via* the PrestoBlue assay, after incubation with PEG nanotubes or MWCNTs for three days (a) (*n* = 4, error bars represent ± standard deviation, * represents statistical significant difference to MWCNTs, (two way ANOVA with Sidak's multiple comparison test (*P* ≤ 0.05))). (b) Brightfield microscope images of human astrocytes after three days of incubation with either PEG nanotubes or MWCNTs (scale bars represent 100 μm).

Carbon nanotubes have been investigated extensively for use in drug delivery applications,^27,45^ but suffer the drawback of inherent cytotoxicity due, in part, to their stiff 1-dimensional structure.^46^ We have previously shown that template synthesised polymer nanotubes are orders of magnitude less stiff than MWCNTs.^24^ We therefore hypothesised that the PEG nanotubes, synthesised herein, would not be cytotoxic to *in vitro* cell cultures. Astrocytes were chosen for initial cell viability analysis because they are a predominant cell type of the brain. We envisaged the development of a delivery system that did not intrinsically cause toxicity but could deliver a chemotherapeutic payload. Fig. 3 confirms that, at the highest concentration analysed (120 μg mL^−1^), the PEG nanotubes did not cause a reduction in the viability of human astrocyte cells (as measured by metabolic activity) in comparison to untreated control cells when analysed after 1 day (Fig. S5†) and 3 days in culture (Fig. 3a). Brightfield images show that PEG nanotube concentrations of 15 μg mL^−1^ (Fig. 3b) and 60 μg mL^−1^ (Fig. S5†) result in a mass of nanotubes over the surface of the cells and well. MWCNTs at these concentrations caused a reduction in cell viability and can be observed as clumps associated with the cells. The lack of cytotoxicity exhibited by the PEG nanotubes reflects our findings *via* breast epithelial cells.^24^

### Drug loaded nanotubes kill glioblastoma cells in 2-dimensional (2D) *in vitro* culture

**Fig. 4:**
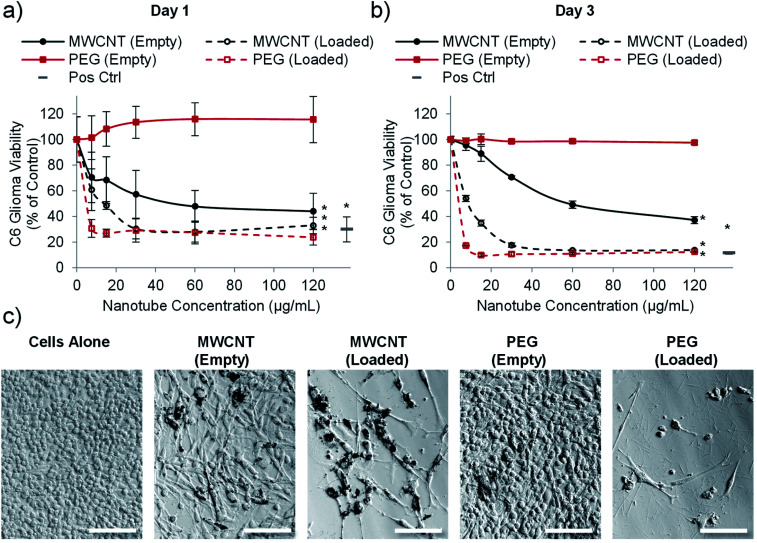
PEG nanotubes only show toxicity when loaded with doxorubicin. C6 glioma cell viability analysis (PrestoBlue assay) after incubation with empty nanotubes or doxorubicin loaded nanotubes (drug to nanotube weight ratio of 0.08 : 1) for one day (a) or three days (b) showing that the PEG nanotubes only reduce C6 glioma viability when loaded with doxorubicin. MWCNTs show dose dependent toxicity which is augmented by doxorubicin loading (*n* = 4, error bars represent ± standard deviation, positive control = 4 μg mL^−1^ doxorubicin, * represents statistical significant difference to empty PEG nanotubes (two way ANOVA with Tukey's multiple comparison test (*P* ≤ 0.05))). (c) Brightfield microscope images of C6 glioma cells after three days of culture showing impaired cell viability in the presence of MWCNTs (empty and loaded) and doxorubicin loaded PEG nanotubes (nanotube concentration = 15 μg mL^−1^, scale bars represent 100 μm, images of the positive control are in Fig. S7†).

**Fig. 5:**
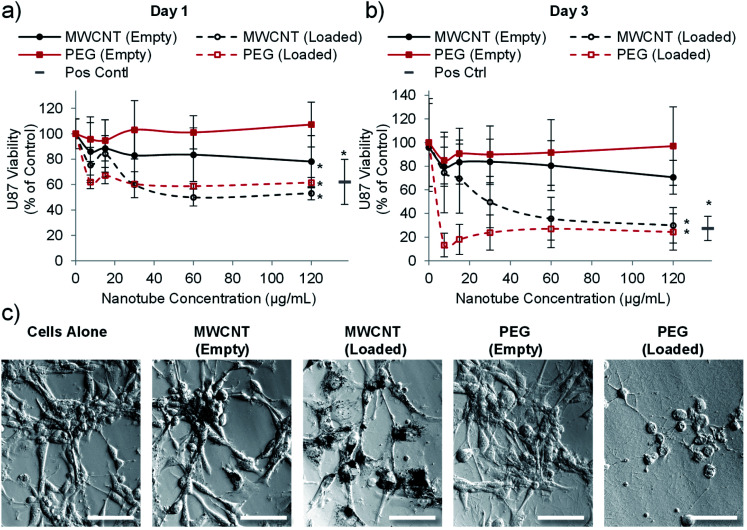
Drug loaded nanotubes reduce the viability of U-87 human glioblastoma cells. Cell viability analysis (PrestoBlue assay) after one day (a) or three days (b) of incubation with PEG or MWCNTs showing that both nanotube types reduce glioblastoma viability when loaded with doxorubicin (*n* = 4, error bars represent ± standard deviation, positive control = 4 μg mL^−1^ doxorubicin, * represents statistical significant difference to empty PEG nanotubes (two way ANOVA with Tukey's multiple comparison test (*P* ≤ 0.05))). (c) Brightfield images taken after three days of culture showing clear association of the MWCNTs with cells whereas PEG nanotubes spread more evenly across the well (nanotube concentration = 15 μg mL^−1^, scale bars represent 100 μm, images of the positive control are in Fig. S7†).

Having established that PEG nanotubes caused no reduction in astrocyte viability at concentrations up to 120 μg mL^−1^, we next wanted to assess the effect of doxorubicin loaded nanotubes on glioma and glioblastoma cell lines. We hypothesised that unloaded (empty) nanotubes would not cause cytotoxicity whereas doxorubicin loaded nanotubes could kill the brain cancer cells, thus indicating that cell death is caused by the released drug. Whilst the PrestoBlue assay, used herein, measures cell metabolic activity, we have used it as an indicator of cell health/viability by normalising to the untreated control group. The viability of C6 glioma cells was analysed 1- and 3-days post incubation with varying concentrations of MWCNT and PEG nanotubes, either empty, or loaded with doxorubicin. In a similar manner to astrocytes, unloaded MWCNTs caused a reduction in C6 cell viability, whilst unloaded PEG nanotubes did not (Fig. 4). Furthermore, both nanotube types caused cytotoxicity, in a dose dependent manner, when loaded with doxorubicin. In general, U-87 glioblastoma cells appeared to be less affected by the drug loaded nanotubes, taking three days to reduce the cell viability below 50% (Fig. 5). However, control cells receiving 4 μg mL^−1^ of free doxorubicin responded similarly, indicating that U-87 cells are perhaps more chemoresistant than C6 cells. A similar study was carried out with U251 malignant glioblastoma cells (Fig. S6†) which followed a similar trend to the U-87 cells. These studies all showed that the unloaded PEG nanotubes did not cause cell death (for all cell types tested), indicating that a non-toxic delivery system can be used to deliver a cytotoxic chemotherapeutic agent.

### Injection of doxorubicin loaded PEG nanotubes into 3-dimensional (3D) cultures of C6 glioma reduces their viability

A previous study using prostate cancer cell lines (LNCaP and PC3 cells) highlighted that matching doses of doxorubicin gave different cytotoxic outcomes depending on whether the cells were cultured in 2D or in 3D (within a hydrogel).^47^ Both cell types exhibited reduced chemosensitivity when cultured in a 3D hydrogel platform.^47^ We therefore wanted to analyse whether drug loaded nanotubes could still exert their cytotoxic effect on cells cultured in a 3D microenvironment. We also wanted to assess the feasibility of injecting the nanotubes, so a Matrigel™-based 3D culture was used as a model to test focal injection and tumour ablation in 3D culture.

**Fig. 6:**
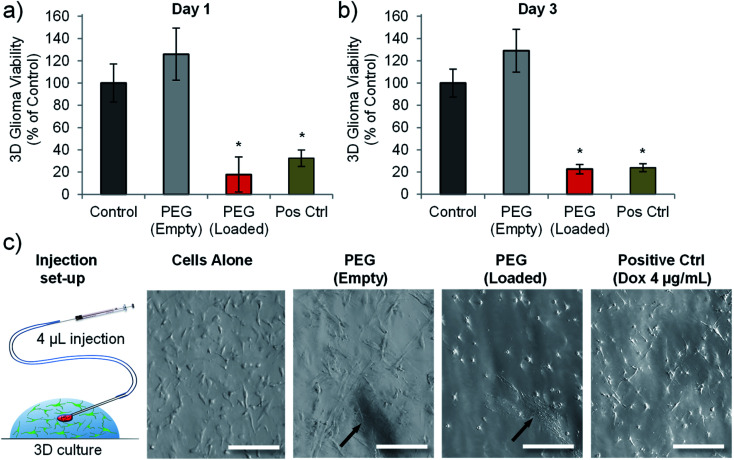
Doxorubicin loaded PEG nanotubes reduced the viability of C6 glioma cells cultured in a 3D environment. Cell viability analysis of C6 glioma cells grown in Matrigel gel-drop culture either one day (a), or three days (b) after injection of either empty PEG nanotubes, or doxorubicin loaded PEG nanotubes (final concentration of 7.5 μg mL^−1^). Cell viability was normalised to untreated cells (control). Doxorubicin treated cells (4 μg mL^−1^) served as a positive control (Pos Ctrl) (*n* = 4, error bars represent ± standard deviation, * represents statistical significant difference to control (one way ANOVA with Tukey's multiple comparison test (*P* ≤ 0.05))). (c) Depiction of injection into the Matrigel model with brightfield microscope images of the cells in 3D culture showing the nanotube injection site (indicated by black arrows) (scale bars represent 100 μm).

Fig. 6 shows that doxorubicin loaded PEG nanotubes, injected into the centre of the 3D culture model resulted in a significant reduction in C6 cell viability down to 23% three days post-injection. This data corresponded well to the cell viability of C6 cells cultured in 2D and incubated with the same concentration of doxorubicin loaded nanotubes (7.5 μg mL^−1^) which had a mean viability of 21% after three days (Fig. 4b). Injection of unloaded nanotubes did not result in a loss of glioma viability. In addition to the PrestoBlue cell metabolic activity assay, live/dead analysis was performed by confocal microscopy (Fig. S8†). This data shows that nanotubes are retained at the injection site and that the doxorubicin-loaded nanotubes caused considerable cell death in the 3D model.

Focal drug delivery, either directly to the tumour, or to the surrounding tissue, represents a means of increasing the local drug concentration in proximity to the tumour mass.^48^ As described earlier, Gliadel wafers have been added to the resection cavity of GBM patients to release carmustine locally to remaining non-resected cancer cells. However, aside from being too big to inject to non-resectable tumours, the flat shape of Gliadel wafers, combined with their stiffness and brittleness, make them poorly matched to the mechanical properties of the brain. Furthermore, Gliadel wafers have been associated with several adverse effects including convulsions, confusion, brain oedema, infection, hemiparesis, aphasia, and visual field defects.^49^ These complications coupled with limited clinical benefits drives the rationale for developing alternative focal drug delivery strategies.^50^ Here, we have demonstrated the cytotoxic efficacy of PEG nanotubes, and the feasibility of injecting them by means of a cannula set-up typically used for intracerebral stereotactic injection into rodents.^51^

### Drug loaded nanotubes reduce glioblastoma multiforme spheroid size and viability

**Fig. 7:**
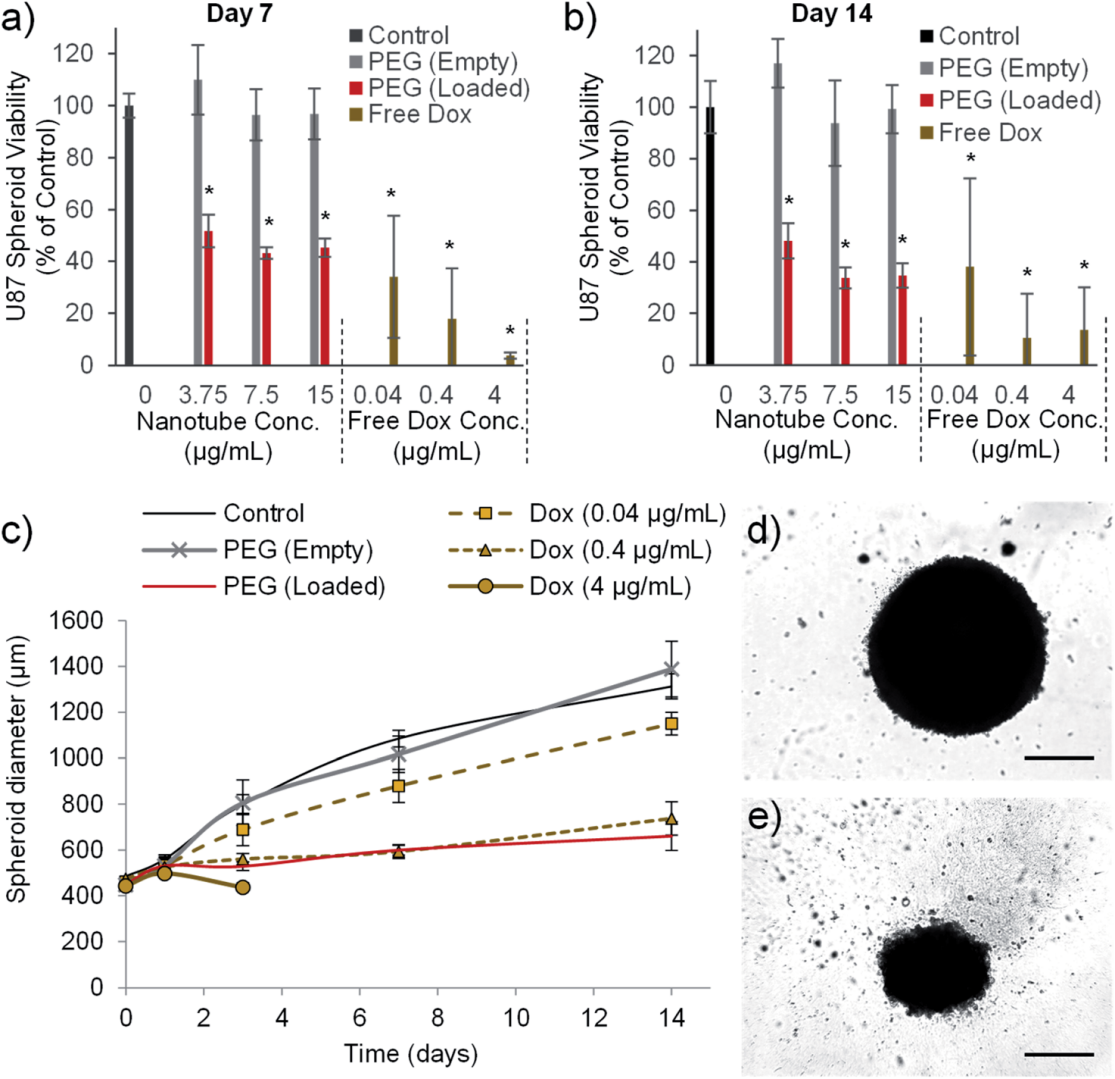
Doxorubicin loaded nanotubes reduce the viability and size of human glioblastoma spheroids. The viability of glioblastoma spheroids formed from 1000 U-87 cells is reduced by doxorubicin and doxorubicin loaded nanotubes, as measured using the PrestoBlue assay after seven days (a) and 14 days (b). Doxorubicin loaded PEG nanotubes (15 μg mL^−1^) mediated a reduction in viability which correlates with a reduction in the growth rate of the spheroid as determined by spheroid diameter (c), with 4 μg of doxorubicin causing complete dissociation of the spheroid after three days in culture (*n* = 6, error bars represent ± standard deviation, * represents statistical significant difference to control (one way ANOVA with Tukey's multiple comparison test (*P* ≤ 0.05))). After 14 days of culture, brightfield microscope images show untreated control spheroids (d) or spheroids cultured with doxorubicin loaded PEG nanotubes (15 μg mL^−1^) (e) (scale bars represent 500 μm).

The final *in vitro* assay used to validate the cytotoxic efficiency of the PEG nanotube drug delivery system utilized U-87 spheroid culture. U8-7 cell spheroid culture more closely recapitulates some aspects of tumour growth such as oxygen/nutrient gradients within the mass of cells,^52^ and has previously been used during the assessment of doxorubicin loaded nanomedicines.^53^ PEG nanotube cytotoxicity was analysed using U-87 cell spheroids cultured over a longer time period (7 and 14 days). Fig. 7 shows that unloaded nanotubes did not cause a reduction in spheroid viability or spheroid size in comparison to untreated spheroids. However, doxorubicin loaded nanotubes resulted in a reduction in viability to less than 52% for all nanotube concentrations analysed. Doxorubicin loaded nanotubes at a concentration of 15 μg mL^−1^ caused a reduction in cell viability to 45% and 35% after 7 and 14 days respectively. This corresponded with a 49% reduction in spheroid diameter compared to the untreated controls after 14 days. It is interesting to note that in the 2D studies with the same cell type (Fig. 5), a doxorubicin loaded PEG nanotube concentration of 15 μg mL^−1^ appeared more cytotoxic, causing a large reduction in viability to 18% already after 3 days.

This discrepancy in cytotoxicity between 3D and 2D culture indicates that U-87 glioblastoma cells cultured as a spheroid may be more robust and less susceptible to anticancer therapeutics than their 2D culture counterpart. In the case of doxorubicin this may be due to a hypoxic region forming at the spheroid core^52^ reducing the cytotoxic action of the drug *via* hypoxia inducible factor 1*α* related pathways.^54,55^ Our study was conducted by adding the nanotubes to the medium surrounding the spheroids which does not recapitulate the process of intratumoural injection.

We then analysed the feasibility of injecting the doxorubicin loaded nanotubes directly into the spheroid core which could more accurately represent the focal drug delivery strategy required for inoperable GBM tumours. This process was technically difficult to accomplish, although loaded nanotubes and free drug could be successfully injected into the spheroids as shown in Fig. S9.† Despite a syringe driver being used to control the injection of nanotubes (0.25 μL) to a rate of 1 μL per minute, not all the injected solution remained within the spheroid. We conclude that this process would require further refinement, both in terms of the approach to injection, and the volumes/rates injected, to yield a viable technique to mimic direct tumoural injection. However, intraspheroid injection of doxorubicin loaded nanotubes could be visualised by fluorescence microscopy, showing retention at the injection site, and a corresponding reduction in viability (Fig. S9g and h†).

The use of doxorubicin in focal/local therapeutic strategies offers potential for improving upon the currently poor prognosis associated with glioblastoma multiforme. Not only has doxorubicin been shown to be more potent against GBM cells in culture than both carmustine^56^ and temozolomide,^14^ but in addition, studies have indicated that a synergistic cytotoxic mechanism exists between doxorubicin and temozolomide.^57,58^ Doxorubicin has also been used clinically for treatment of GBM in conjunction with temozolomide *via* pegylated liposomal doxorubicin,^59^ or the EnGeneIC delivery vehicle.^60^ These delivery systems have been designed for systemic administration and the lack of improvement shown by these trials over standard therapeutic strategies may in part be due to a low concentration of the drug reaching the target site. In order to maximise the potential for doxorubicin based strategies, injectable local drug delivery systems need to be explored.^61^

Whilst the data herein does not show that doxorubicin-loaded nanotubes are more effective than the free drug, these *in vitro* models fail to recapitulate the complex nature of drug dispersion, elimination and termination *in vivo*, where a termination half-life for doxorubicin of under 48 hours can be expected.^62,63^ Local drug delivery systems may therefore negate the need for continuous or repeat administration to the tumour site.

## Conclusions

In summary, we have used a template-assisted synthesis strategy to produce PEG-based nanotubes. The ability of the nanotubes to be loaded with doxorubicin could be tuned simply by varying the template dissolution time (and hence the degree of carboxylic acid functionality). Doxorubicin was readily loaded to the negatively charged PEG nanotubes *via* electrostatic interaction with the positively charged (protonated) doxorubicin. The loaded PEG nanotubes showed a slow release of doxorubicin over 21 days with the majority released over the first week. In contrast to stiff MWCNT used as a reference, the unloaded flexible PEG nanotubes showed good biocompatibility across all cell types tested whilst the doxorubicin loaded nanotubes reduced GBM cell viability (C6, U-87 and U-251) in a dose dependent manner in 2D and 3D models. Focal administration of these injectable polymer nanotubes therefore holds the potential to broaden the spectrum of anti-cancer drugs available for GBM therapies.

## Conflicts of interest

There are no conflicts to declare.

## Supplementary Material

NA-002-D0NA00471E-s001
