# Supplementary material for: Poly(ethylene glycol) based nanotubes for tuneable drug delivery to glioblastoma multiforme
Source: Nanoscale Adv. 2020 Aug 24;2(10):4498–509. doi: 10.1039/d0na00471e (PMC9418774; doi:10.1039/d0na00471e)
Supplement: NA-002-D0NA00471E-s001 [file NA-002-D0NA00471E-s001.pdf]

Electronic Supplementary Material (ESI) for Nanoscale Advances.
This journal is © The Royal Society of Chemistry 2020

Poly(ethylene glycol) based nanotubes for tuneable drug delivery to glioblastoma multiforme

Majed Alghamdi, Filippo Chierchini, Dimitri Eigel, Christian Taplan, Thomas Miles, Dagmar Pette, Petra B. Welzel, Carsten Werner, Wenxin Wang, Catia Neto, Mark Gumbleton and Ben Newland,*

Supplementary Information

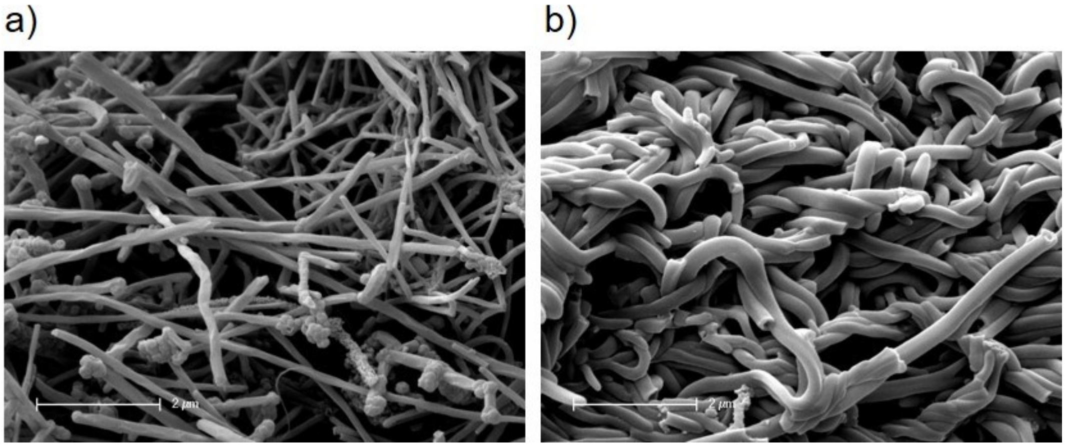

Figure S1 – Scanning electron microscope images of multiwalled carbon nanotubes (MWCNT) **(a)** in comparison to the PEG nanotubes **(b)** taken at the same magnification (scale bars represent 2 μm). Here the wide variation in diameter of the MWCNT can be clearly observed.

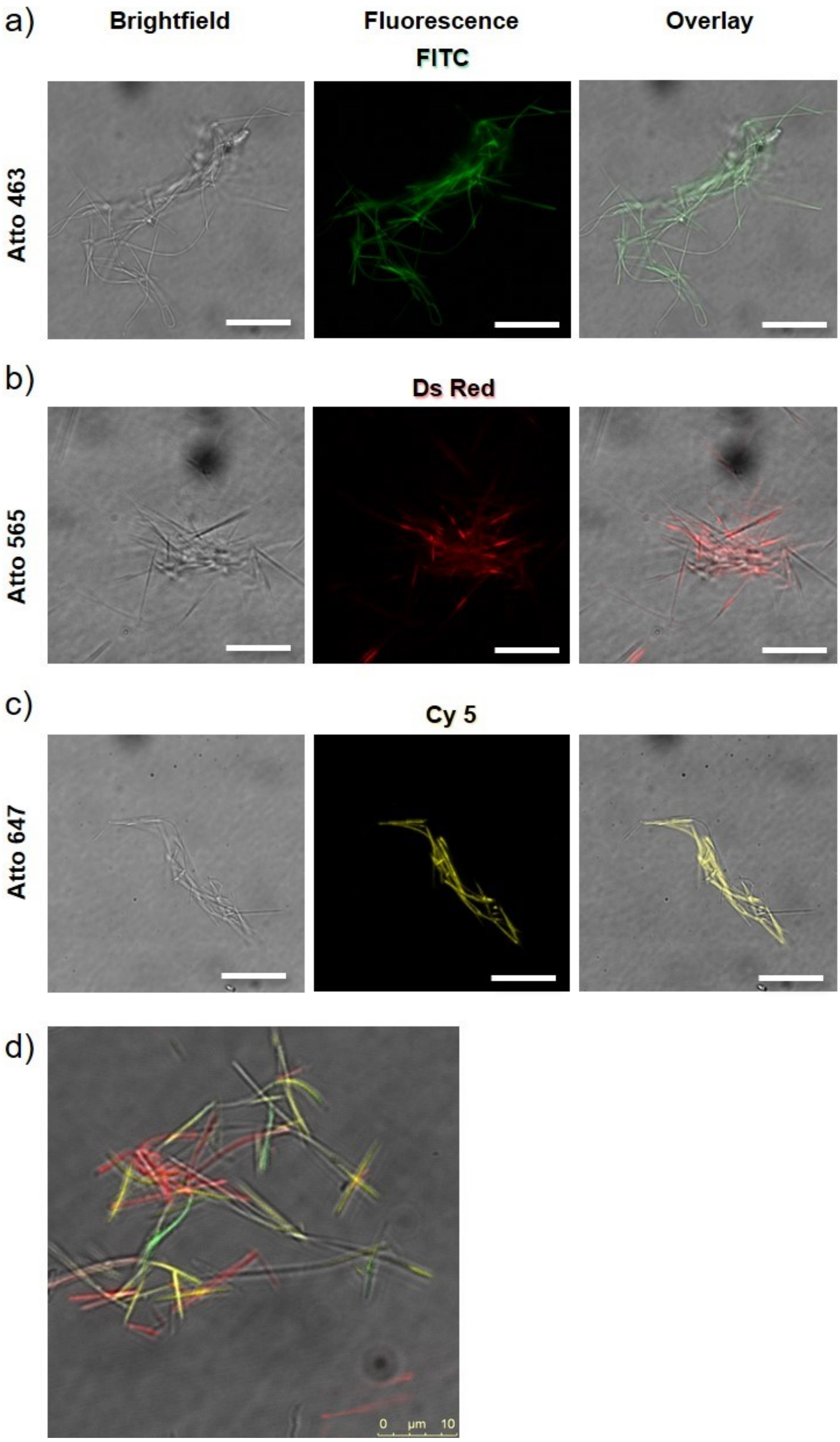

Figure S2 – Fluorescent labelling of PEG nanotubes can be performed with maleimide functionalised Atto dyes 463 **(a)**, 565 **(b)** and 647 **(c)** allowing fluorescent microscope detection using different excitation and emission wavelengths (scale bars represent 20 µm), as exemplified with a mix of PEG nanotubes **(d)** (scale bar represents 10 µm).

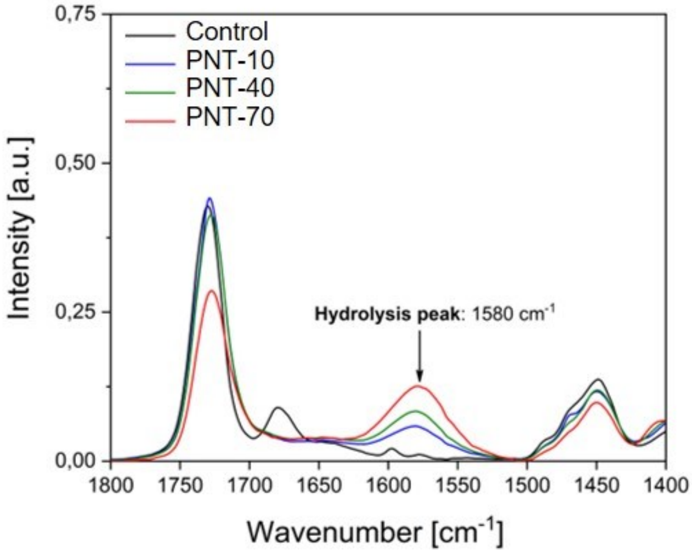

Figure S3 – Raman spectroscopy analysis of PEG nanotubes after 10, 40 and 70 minutes of template dissolution in sodium hydroxide, showing a rise in the peak at 1580 cm^{-1} indicating hydrolysis of the ester bond to a carboxylic acid. The control was crosslinked poly(ethylene glycol) diacrylate that had not been exposed to sodium hydroxide.

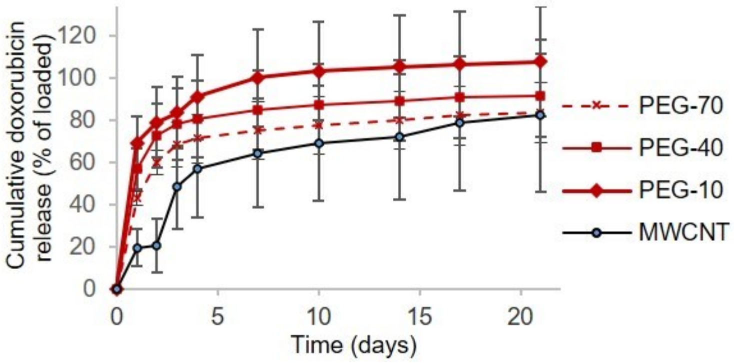

Figure S4 – Cumulative doxorubicin release from Figure 2 in the main text, expressed as a percentage of drug loaded. Although the majority of the drug is released from the nanotubes by day seven, there is still a small amount released over the following 14 days (n=4, error bars represent ± standard deviation).

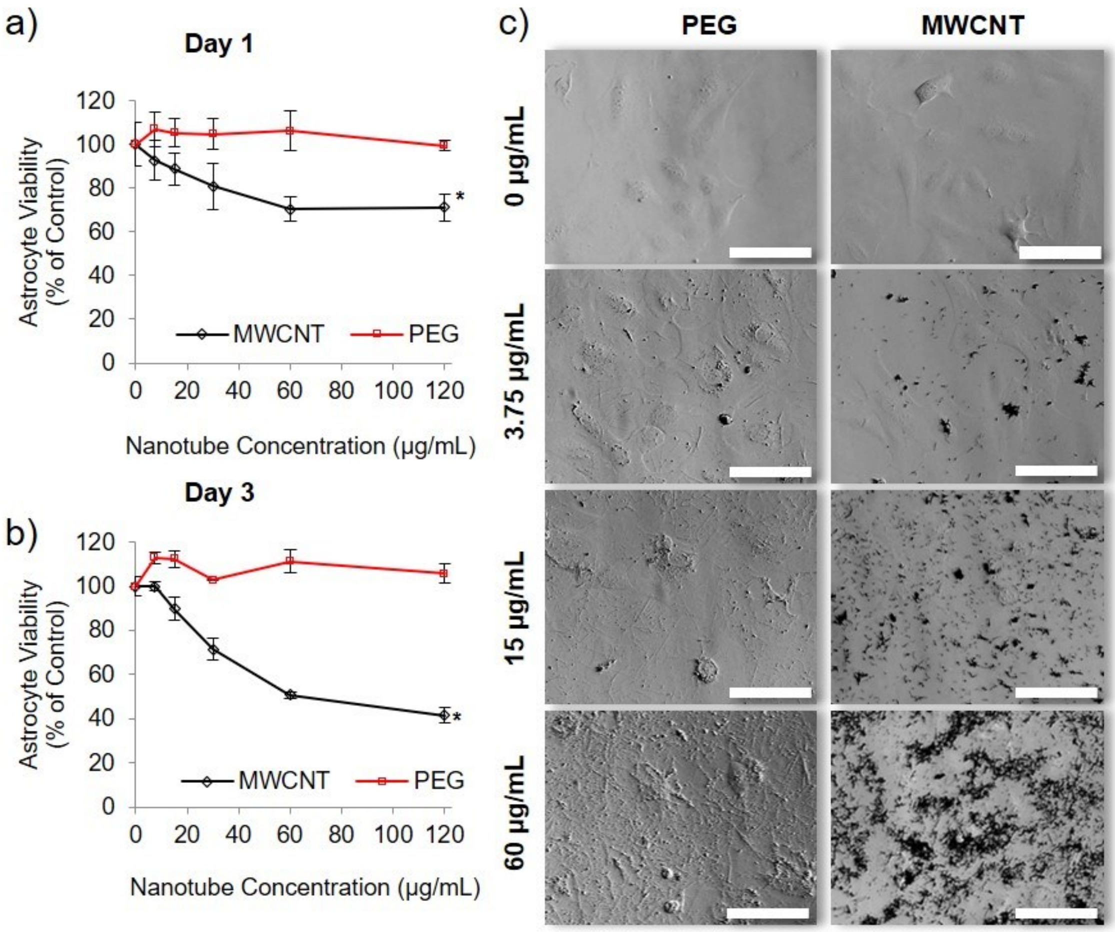

Figure S5 – Additional data for Figure 3 of the main text. Empty PEG nanotubes show significantly lower toxicity than MWCNT. The viability of human astrocytes, as determined via the PrestoBlue assay, after incubation with PEG nanotubes or MWCNTs for one day **(a)** or three days **(b)** (n=4, error bars represent ± standard deviation, * represents statistical significant difference to MWCNTs, (two way ANOVA with Sidak's multiple comparison test (P ≤ .05))). **c)** Brightfield microscope images of human astrocytes after three days of incubation with either PEG nanotubes or MWCNTs (scale bars represent 100 µm).

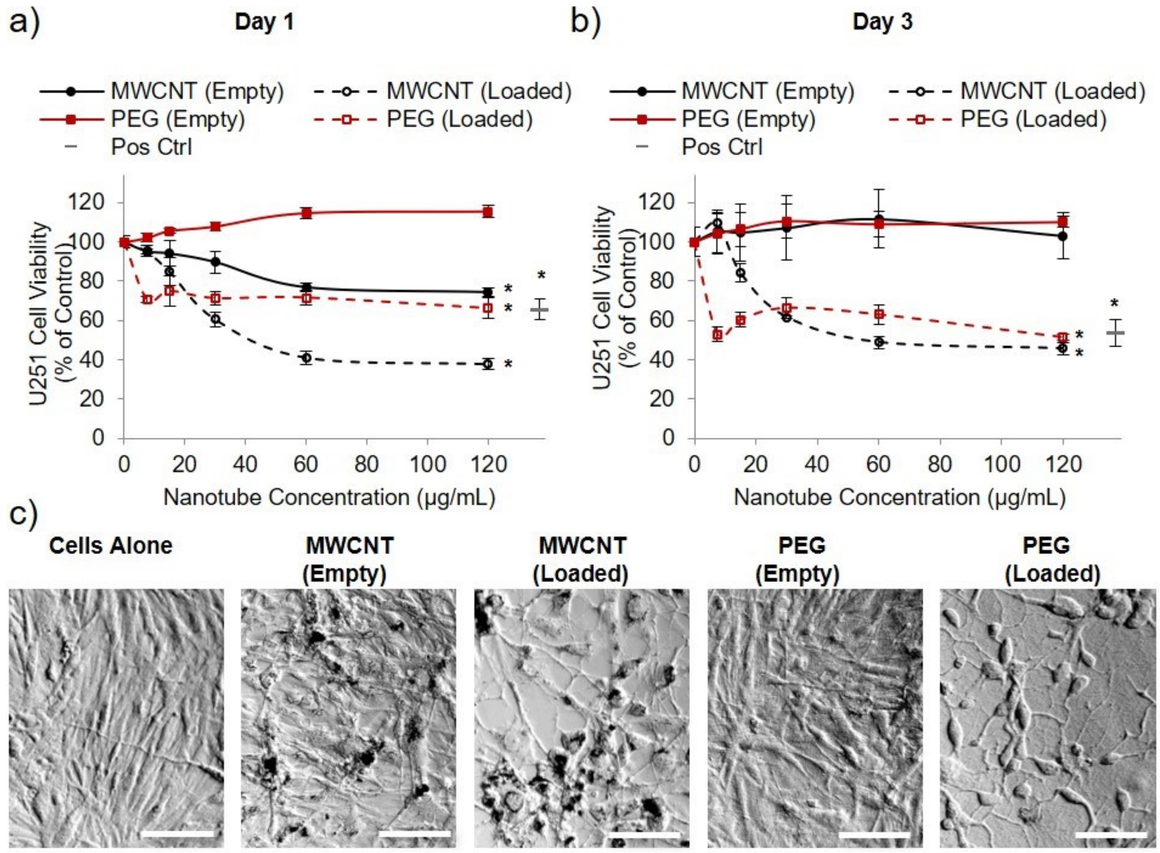

Figure S6 – Drug loaded nanotubes reduce the viability of U251 MG human malignant glioblastoma multiforme cells. Cell viability analysis (PrestoBlue assay) after one day **(a)** or three days **(b)** of incubation with PEG or MWCNTs showing that both nanotube types reduce glioblastoma viability when loaded with doxorubicin (n=4, error bars represent ± standard deviation, positive control = 4 µg/mL doxorubicin). **c)** Brightfield images of the cells after three days of culture showing a clear change in cell morphology after incubation with drug loaded nanotubes (nanotube concentration = 15 µg/mL, scale bars represent 100 µm).

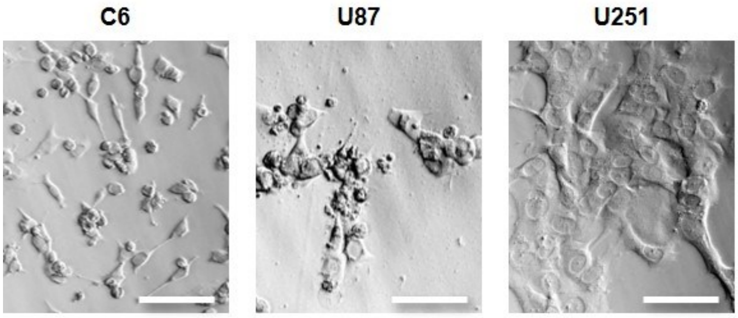

Figure S7 – Brightfield microscope images of the cells that received the positive control (4 µg/mL doxorubicin) for three days in culture, in association with main text figure 4 (C6 cells), figure 5 (U87 cells) and supplementary figure S6 (U251 cells) (scale bars represent 100 µm).

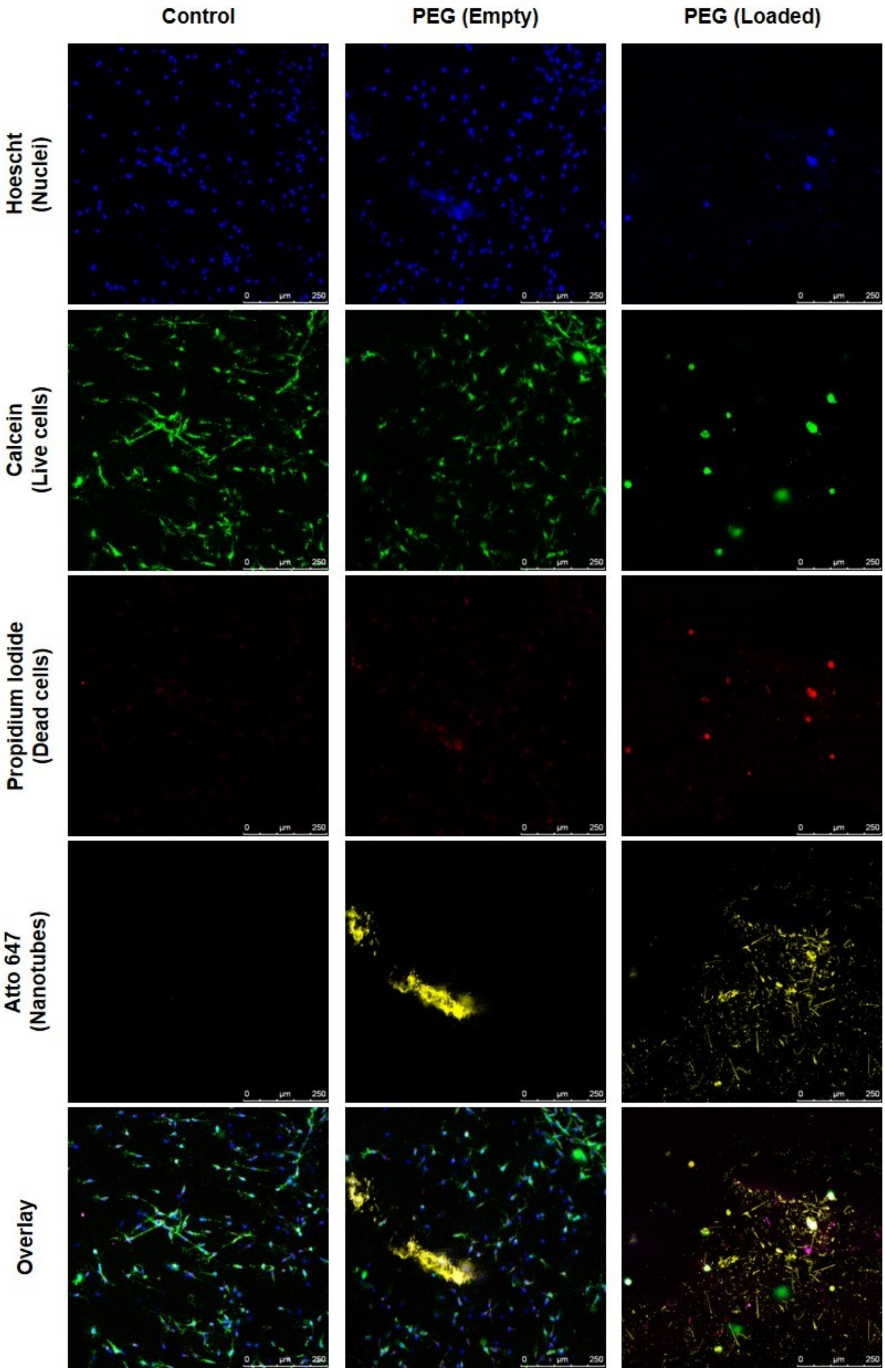

Figure S8 – Representative confocal microscope images showing the projection average of a 30 µm Z-stack through the C6 glioma cells cultured in Matrigel three days post-injection of either empty or doxorubicin-loaded nanotubes as per Figure 6 of the main text. These images show that the Atto-labelled nanotubes are retained in relatively focal position post-injection. Scale bars represent 250 µm.

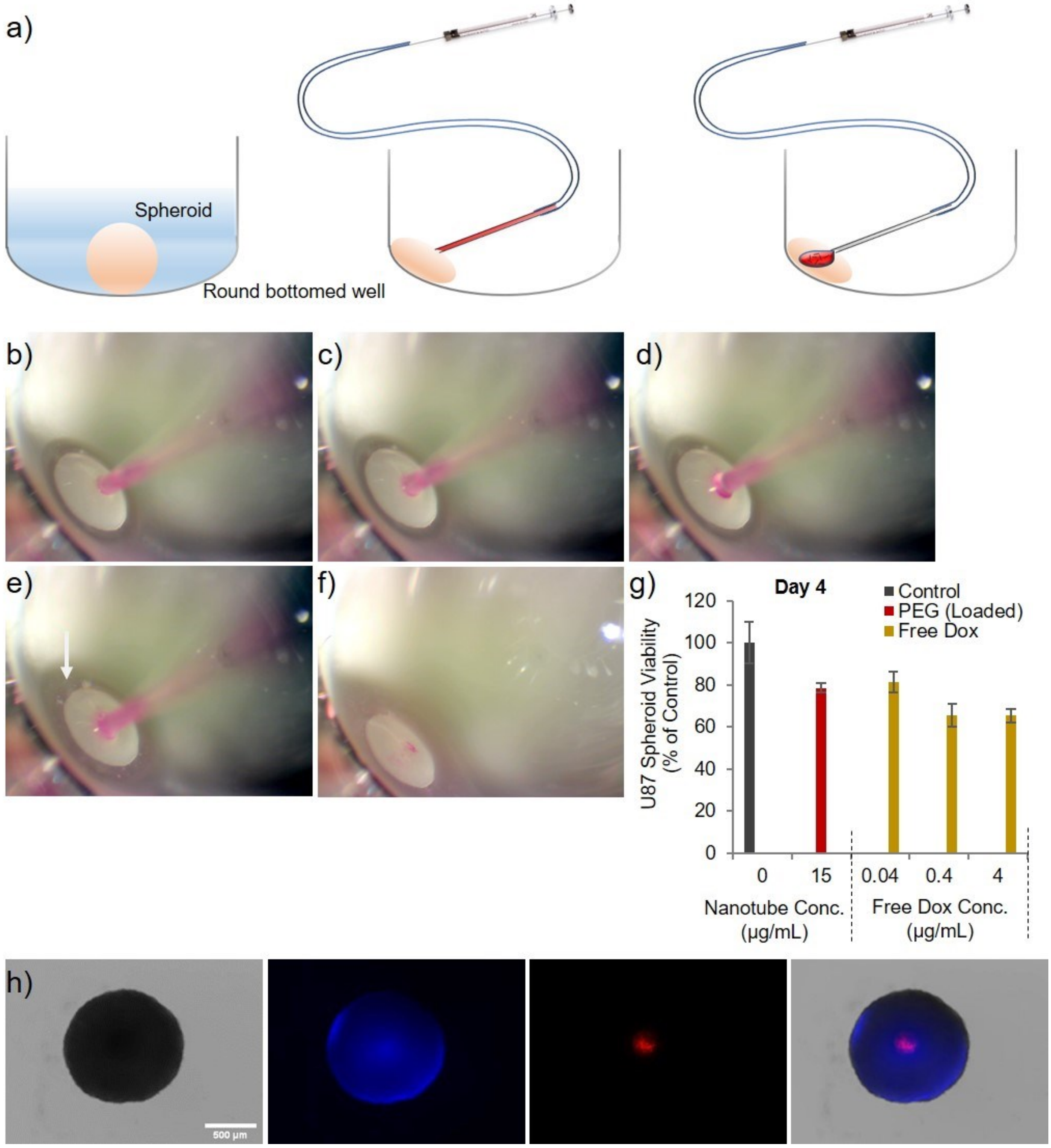

Figure S9 – A schematic depiction of the process of injecting glioblastoma spheroids (a), showing the removal of the culture medium, moving of the spheroid to the side of the well, and careful injection into the centre. Images **(b – f)** show the process of injection including the approach (b), penetration (c), initiation of injection (d), then some spill out from the spheroid (indicated by the white arrow) (e) and the resulting spheroid prior to the replacement of the medium (f). Presto blue analysis of doxorubicin-loaded nanotubes in comparison to free doxorubicin, 4 days post injection (n=4, error bars represent +/- standard deviation). Fluorescent microscope analysis shows a spheroid stained with Hoescht nuclear stain (blue) and the fluorescence of doxorubicin (red) from the doxorubicin loaded nanotubes post-injection with subsequent overlay (scale bar represents 500 µm).
